# Influenza A penetrates host mucus by cleaving sialic acids with neuraminidase

**DOI:** 10.1186/1743-422X-10-321

**Published:** 2013-11-22

**Authors:** Miriam Cohen, Xing-Quan Zhang, Hooman P Senaati, Hui-Wen Chen, Nissi M Varki, Robert T Schooley, Pascal Gagneux

**Affiliations:** 1 Department of Cellular and Molecular Medicine, University of California San Diego, 9500 Gilman Dr, La Jolla 92093, California, USA; 2 Division of Infectious Disease, University of California San Diego, 9500 Gilman Dr, La Jolla 92093, California, USA; 3 Present address: School of Veterinary Medicine, National Taiwan University, 1 Sec. 4 Roosevelt Rd, Taipei 10617, Taiwan

**Keywords:** Influenza A, Sialic acids, Mucus, Neuraminidase, Infection, Saliva

## Abstract

**Background:**

Influenza A virus (IAV) neuraminidase (NA) cleaves sialic acids (Sias) from glycans. Inhibiting NA with oseltamivir suppresses both viral infection, and viral release from cultured human airway epithelial cells. The role of NA in viral exit is well established: it releases budding virions by cleaving Sias from glycoconjugates on infected cells and progeny virions. The role of NA in viral entry remains unclear. Host respiratory epithelia secrete a mucus layer rich in heavily sialylated glycoproteins; these could inhibit viral entry by mimicking sialylated receptors on the cell surface. It has been suggested that NA allows influenza to penetrate the mucus by cleaving these sialylated decoys, but the exact mechanism is not yet established.

**Methods:**

We tested IAV interaction with secreted mucus using frozen human trachea/bronchus tissue sections, and bead-bound purified human salivary mucins (HSM) and purified porcine submaxillary mucins (PSM). The protective effect of mucus was analyzed using MDCK cells coated with purified HSM and PSM with known Sia content. Oseltamivir was used to inhibit NA activity, and the fluorescent reporter substrate, 4MU-Neu5Ac, was used to quantify NA activity.

**Results:**

IAV binds to the secreted mucus layer of frozen human trachea/bronchus tissues in a Sia dependent manner. HSM inhibition of IAV infection is Sia dose-dependent, but PSM cannot inhibit infection of underlying cells. HSM competitively inhibits NA cleavage of 4MU-Neu5Ac, reporter substrate. Human IAV effectively cleaves Sias from HSM but not from PSM, and binds to HSM but not to PSM.

**Conclusion:**

IAV interacts with human mucus on frozen tissue sections and mucus-coated beads. Inhibition of IAV infection by sialylated human mucus is dose-dependent, and enhanced when NA is inhibited with oseltamivir. Thus NA cleaves sialylated decoys during initial stages of infection. Understanding IAV interactions with host mucins is a promising new avenue for drug development.

## Background

Interactions of Influenza A viruses (IAVs) with mucus were first described in the mid 20^th^ century, and led early researchers to classify influenza as a (ortho)myxovirus – a virus with affinity for mucus [1,2]. IAVs must penetrate a secreted mucus layer (up to 50 μm thick) to reach target tissues in mammalian airways [3]. Mucus is a defensive layer containing highly glycosylated mucins rich in terminal sialic acids (Sias) [4]. It has been suggested that mucus may protect against IAV infection by presenting sialylated “decoys” that mimic receptors on the cell surface [5,6]. Influenza viruses bind these unproductive receptors, become trapped in the mucus layer, and can then be removed by the normal process of mucus clearance as part of the innate defense system [7,8].

Neuraminidase (NA) enzymatic activity cleaves Sias from glycoconjugates on infected cells and progeny virions allowing budding virions to escape from infected cells. NA inhibitors such as oseltamivir inhibit viral release by preventing the cleavage of Sias specifically [9]. It has also been shown that NA inhibition with oseltamivir carboxylate suppresses IAV infection of cultured human airway epithelium (HTBE) [10]. Thus NA activity must have a role during initial infection, but the exact mechanism is not yet known. Since HTBE cells are coated with a secreted mucus layer, it was speculated that NA activity promotes infection by cleaving Sias from mucus [10]. However, other interpretations such as promotion of hemagglutinin-mediated fusion [11,12] cannot yet be ruled out, and IAV interactions with mucins during initial infection remain poorly characterized.

Mucin glycosylation and sialylation vary significantly between species, and thus could influence influenza host species specificity. Human airway and salivary mucins have been well characterized [13-15] and their constituents and glycosidic linkages differ dramatically from those of other species such as chimpanzees [16] and pigs [17]. Humans express predominantly *N*-acetylneuraminic acid (Neu5Ac) [18]. Human airway sialoglycans lack the sialic acid (Sia) *N*-glycolylneuraminic acid (Neu5Gc), which is the predominant Sia in porcine mucus [17]. In the human upper respiratory tract Sias are predominantly found in α2-6 glycosidic linkage [19]. In contrast, these Sias are mostly α2-3-linked in chimpanzees [16] and pigs [20].

Mucus carries a vast array of variable receptors, and mucin sialylation can create sites in the mucus that resemble the target receptors on cells [16,21]. IAV binding properties are important determinants of host susceptibility and host range, and the type and distribution patterns of sialylated glycans on target tissues seem to be crucial [22]. Notably, binding properties of IAV are traditionally studied in absence of secreted mucins despite the fact that all natural infection sites are abundantly covered with these secreted sialoglycoproteins.

In this study we investigate the interaction of IAV with mucins, and provide experimental evidence for the role of NA during initial infection of mucus-coated cells and tissues. We demonstrate Sia-dependent binding of IAV to secreted mucus on frozen human trachea/bronchus tissues. We present direct *in vitro* evidence that secreted mucus protects underlying cells from infection by presenting sialylated decoys for hemagglutinin (HA) and competing for NA cleavage activity. We used purified mucus from two different hosts: human and pig, to show direct cleavage of- and direct binding to- sialylated human mucus by human IAV NA and HA, respectively.

## Results

### Influenza A virus interacts with mucin on human airway tissue

**Figure 1:**
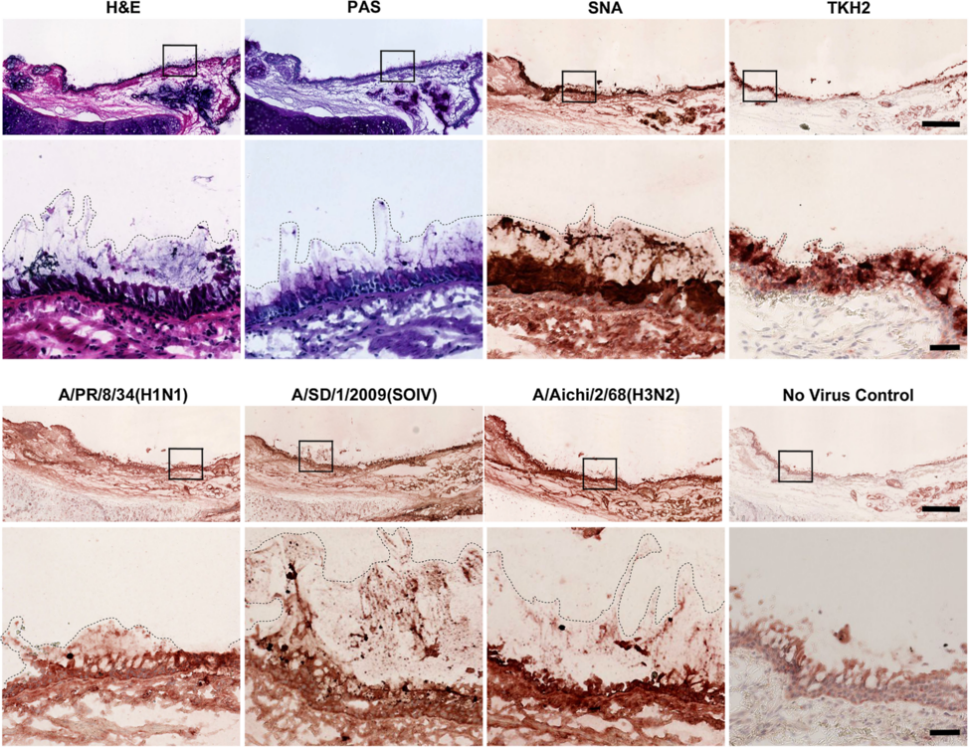
**IAV binds to secreted mucus in human trachea tissues.** Frozen human trachea tissue sections were stained with Hematoxylin and Eosin (H&E), periodic acid Schiff (PAS, mucin staining in pink), *Sambucus nigra* agglutinin (SNA, binds to Siaα2-6Gal/GalNAc) or TKH2 antibody (binds to Sialyl Tn: Siaα2-6GalNAc on *O*-linked glycans). Dashed lines specify the location of secreted mucus, which is preserved in the frozen tissues, as seen in H&E and PAS staining. Both SNA and TKH2 bound to the secreted mucus (dark brown staining), indicating abundant potential ligands for IAV binding. TKH2 binding was confined to the lining of the epithelium and the glands, further confirming that the secreted mucus layer is adequately preserved in these tissues. Binding of IAV to the tissues was tested by incubating 600 HAU of virus 1.5 h at room temperature. All three strains bound to the secreted mucus layer (dashed line), and to ciliated cells (cells stained in dark brown). Virus was detected by anti-NP antibodies. Boxed area is enlarged below each image. Scale bar indicates 500 μm, scale bar of enlarged area indicates 50 μm.

**Figure 2:**
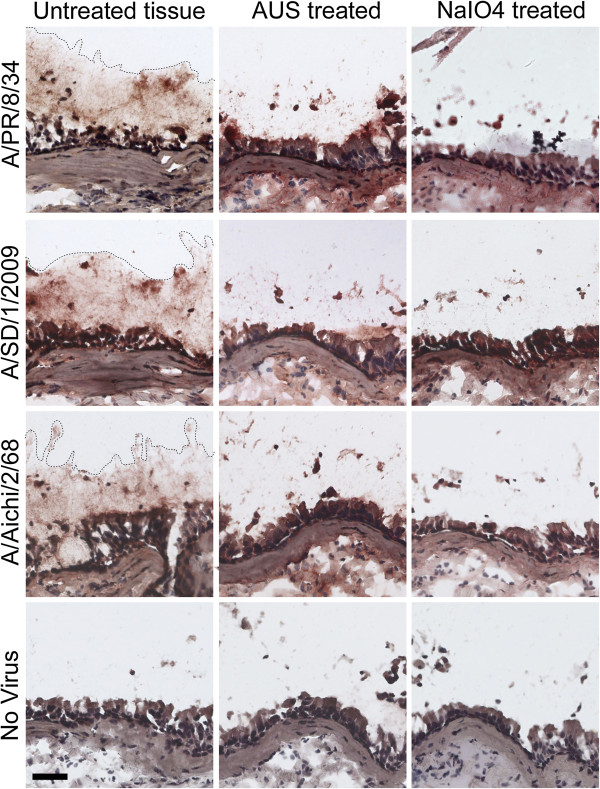
**IAV binding to secreted mucus is Sia-dependent.** Human trachea tissue sections were treated with *Arthrobacter ureafaciens* sialidase (AUS), which cleaves Sias, or with mild sodium periodate (NaIO4), which truncates the Sia side chain. Both treatments reduce IAV binding to the secreted mucus on human trachea tissues compared to untreated control tissues, confirming that IAV binding to the secreted mucus is Sia-dependent. Dashed lines specify location of virus binding to secreted mucus. Scale bar indicates 50 μm.

IAV tropism depends on HA binding specificity and the host sialylation pattern. The distribution of terminal Sias in α2-6 and in α2-3 linkages varies along the respiratory tract, and changes with age and developmental stage [19,23]. Human respiratory tract sialylation patterns have been extensively studied on paraffin embedded tissues, which are lacking much of the secreted mucus layer [23,24]. Here we examine glycosylation and IAV binding to frozen human trachea/bronchus tissues that were frozen and embedded in optimal cutting temperature (OCT) compound. This treatment preserves the secreted mucus layer in a natural state, enabling both immunohistochemistry and virus binding studies [25]. Secreted mucus forms a visible lining on the epithelium of human bronchial tissues, detected by Periodate Acid Schiff staining (Figure 1, PAS, dashed line indicates secreted mucus). Potential receptors for human IAV on secreted airway mucus were detected with *Sambucus nigra* lectin (SNA), which binds to Siaα2-6Gal/GalNAc, or with TKH2 antibody, which bind to Siaα2-6GalNAc on O-linked glycans (Sialyl Tn) (Figure 1, SNA & TKH2, outlined dark brown staining). Sialyl Tn is a glycan epitope that is abundant on mucins but infrequent in other tissues [26]. TKH2 staining is confined to the secreted material lining the epithelium and the glands (Figure 1, TKH2), further confirming that this material represents the secreted mucus layer. In order to test the ability of IAV to bind secreted mucus, these tissues were incubated with 600 HAU of two seasonal virus strains, A/PR/8/34(H1N1) and A/Aichi/2/68(H3N2), and a clinical isolate of the pandemic A/SD/1/2009(SOIV). All three virus strains bound to secreted mucus as well as to the underlying ciliated cells (Figure 1, lower panels, dashed lines). Removal of Sias from the tissues by enzymatic cleavage with *Arthrobacter ureafaciens* sialidase (Figure 2, AUS) significantly reduces virus binding to the mucus, confirming specific binding to sialylated receptors. Similarly, truncation of the Sia side chain by mild sodium periodate treatment [27] reduces virus binding to the mucus (Figure 2, NaIO4). These findings confirm that the secreted mucus layer presents sialylated decoy receptors for binding by IAV and other pathogens.

### Human mucin protects cells from infection *in vitro*

**Figure 3:**
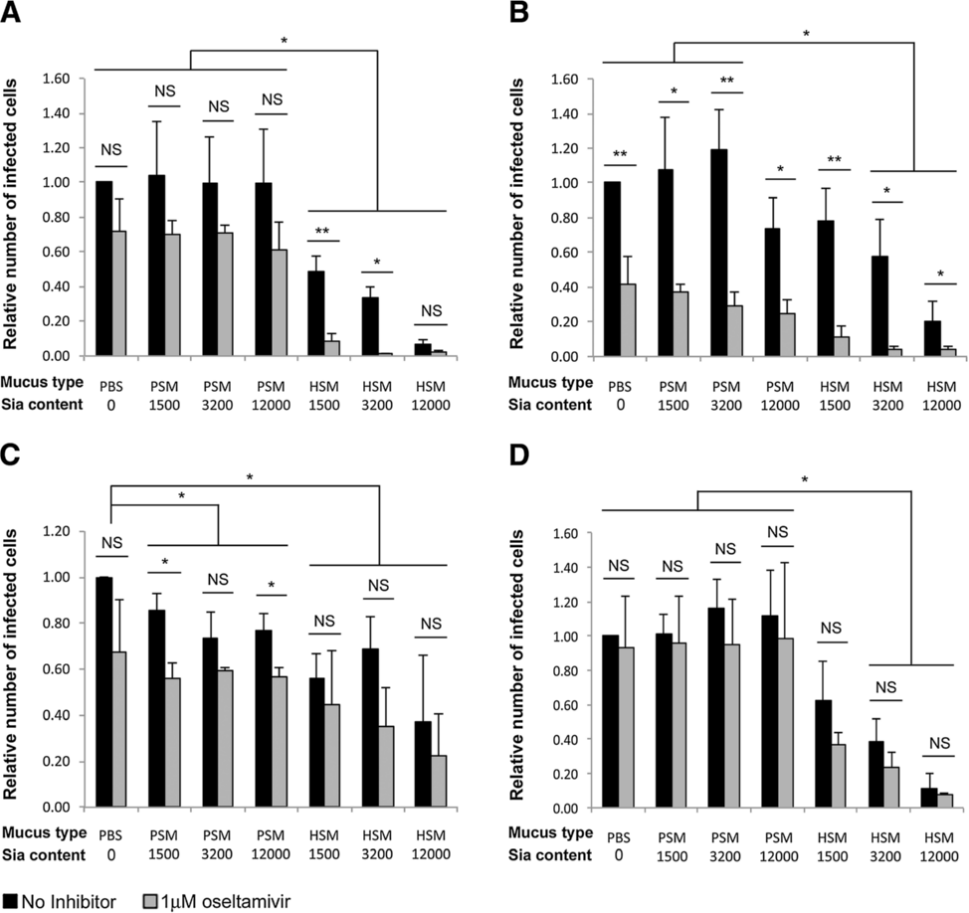
**HSM and oseltamivir have additive inhibitory effects.** MDCK cells were layered with PSM or HSM at 1,500, 3,200, 12,000 pmol Sia/well or with PBS buffer as control. The cells were challenged for 1h at 37°C with 10^9^ TCID_50_ of **(A)** A/PR/8/34(H1N1), **(B)** A/SD/1/2009(H1N1), **(C)** A/SD/17/2008(H1N1), or **(D)** A/Aichi/2/68(H3N2) in the presence (gray bars) or absence (black bars) of 1 μM oseltamivir. Infected cells were identified by staining with anti-NP antibodies, and quantified in twenty randomly selected images from each sample. Experiments were repeated three times, for each experiment the number of infected cells in the PBS-coated sample was set to 1, and the relative number of infected cells for each treatment was calculated. Lower number of infected cells was observed in HSM coated monolayers compared to PBS-coated monolayers for all tested virus strains (**A-D**, P<0.05). Dose effects of Sia content in HSM-coated samples were observed for three IAV strains **(A-B, D)**. Significant reduction in the number of infected cells in PSM-coated monolayers was observed only for one strain (**C**, P<0.05). With exception of the A/SD/1/2009(H1N1) strain, oseltamivir did not inhibit infection of cell coated with either PBS or PSM (**A, C-D**, gray bars). In contrast, addition of oseltamivir to HSM-coated samples further reduced infection of A/PR/8/34(H1N1), A/SD/1/2009(H1N1) strains **(A-B)**. Both of the oseltamivir-insensitive strains, A/SD/17/2008(H1N1) and A/Aichi/2/68(H3N2), were not affected by addition of the drug **(C-D)**. Data was analyzed by 3-way ANOVA, corrected for multiple comparisons using Tukey’s HSD (see Additional file 2 for complete statistical analysis data). Error bars represent standard deviation. *P<0.05, **P<0.005.

Since IAV can both bind and cleave sialylated epitopes, we tested whether sialylated mucins can effectively protect underlying cells from IAV infection *in vitro*. Confluent monolayers of MDCK cells in 16-well Lab-Tek chamber slides were overlaid with human salivary mucins (HSM), porcine submaxillary mucins (PSM) or buffer. The mucin content of HSM is similar to that of human airway epithelium submucosal glands (Additional file 1A) [15]. HSM preparation is enriched by acid precipitation of mucins and filtration of saliva samples [28] (Additional file 1B-C). Thus the HSM preparation is a good representation of the mucus of human upper respiratory system. The Sia contents of HSM and PSM samples were determined by DMB-HPLC (Additional file 1D), and MDCK cells were overlaid with mucus containing a known amount of Sias. Total Sia content in the wells was 12,000 pmol/well (high), 3,200 pmol/well (medium) or 1,500 pmol/well (low). The protection efficacy of HSM and PSM against IAV infection of the underlying cells was determined by challenging the cells with 10^9^ TCID_50_ of four IAV strains: A/PR/8/34(H1N1), A/SD/1/2009(SOIV), A/SD/17/2008(H1N1), and A/Aichi/2/68(H3N2) for 1 h at 37°C. Cells were then washed to remove both virus and mucus, and fresh DMEM-TPCK media was added. The cells were incubated for additional 5.5 h at 37°C, fixed and stained for viral nuclear proteins. The number of infected cells was quantified in twenty randomly selected images from each sample, and the infection rate relative to buffer coated cells was determined (Figure 3). For all four IAV strains, coating of cells with HSM at medium or high Sia content significantly reduces the infection of underlying cells compared to buffer coated cells (Figure 3, compare PBS (0) to HSM (3200) and HSM (12000 pmol), *P*<0.05). Only three of the tested IAV strains were significantly inhibited by HSM at low Sia content: A/PR/8/34(H1N1), A/SD/17/2008(H1N1) and A/Aichi/2/68(H3N2) (Figure 3A and C-D, compare PBS (0) to HSM (1500 pmol), *P*<0.05). Notably a dose effect of Sia-content in the HSM layer was observed for three of the IAV strains (A/PR/8/34(H1N1), A/SD/2009(SOIV) and A/Aichi/2/68(H3N2)) where higher Sia-content resulted in fewer infected cells. The numbers of infected cells were 60-95% lower in monolayers coated with HSM (high), 40-65% lower in monolayers coated with HSM (medium), and 40-50% lower in monolayers coated with HSM (low), depending on the strain (Figure 3A-B and D, see Additional file 2 for complete statistical analysis). In contrast to HSM, coating cells with PSM typically did not result a significant reduction in the number of infected cells (Figure 3A-B and D). However, a mild reduction (15-25%) in number of cells infected by A/SD/17/2008(H1N1) was observed in PSM coated monolayers (Figure 3C, *P*<0.05).

High Sia-content PSM (high) is comprised of 10,200 pmol Neu5Gc and 1,800 pmol Neu5Ac (Additional file 1D). However, the presentation of Sia differs between PSM [17] and HSM [13-15]. Despite having a similar Neu5Ac content, PSM (high) and HSM (low) did not have the same inhibitory effect on IAV infection. Two strains, A/PR/8/34(H1N1) and A/Aichi/2/68(H3N2) were not significantly inhibited by coating monolayers with PSM (high), however coating monolayers with HSM (low) significantly reduced the number of infected cells (Figure 3A and D, *P*<0.0005, and *P*<0.0462, respectively). Numbers of cells infected by A/SD/17/2008(H1N1) were reduced in monolayers coated with both PSM (high) and HSM (low), however, fewer infected cells were observed in HSM-coated monolayers compared with PSM-coated monolayers (Figure 3C, *P*<0.0504). In contrast coating monolayers with either PSM (high) or HSM (low) did not significantly reduce the number of cells infected with A/SD/1/2009(SOIV) (Figure 3B). Thus Sia content, type, and presentation are all important factors for inhibition of IAV infection. Furthermore, mucus inhibition of IAV infection is strain-dependent.

### Inhibition of IAV neuraminidase by oseltamivir increases the protective effect of HSM but not PSM

Since the virus NA can potentially cleave sialylated receptors presented on secreted human mucus, inhibition of NA activity may enhance the protective effect of mucus. In order to test this hypothesis, 1 μM oseltamivir was added to IAV prior to challenging the mucus-coated cells. The most prominent reduction of infection rate by oseltamivir was observed for the pandemic A/SD/2009(SOIV) strain. A reduction of ~60% in the number of infected cells was observed in monolayers coated with either buffer or PSM (Figure 3B, PBS and PSM *P*<0.05). This could be attributed to oseltamivir inhibition of the secondary Sia-binding site found on the virus N1 neuraminidase [29], rather than to the neuraminidase enzymatic activity. However, the number of infected cells in HSM-coated monolayers was further reduced to <10% upon addition of oseltamivir, even with monolayers that were coated with HSM (low). Thus oseltamivir and HSM have an additive inhibitory effect (HSM + oseltamivir compared with HSM, or with PBS + oseltamivir, *P*<0.05, see Additional file 2 for complete statistical analysis). The number of infected cells in PSM (high)-coated monolayers and HSM (low)-coated monolayers were reduced to a similar extent by the addition of 1 μM oseltamivir (Figure 3B, 24±8% and 11±7%, respectively, *P*<0.0853).

Similar results were obtained for the seasonal H1N1 strain (A/PR/8/34(H1N1)). Although the number of infected cells in PSM- or PBS-coated monolayers was not significantly reduced by oseltamivir, 25-30% reduction in the number of infected cells was observed (Figure 3A, gray bars). However, the number of infected cells in HSM-coated monolayers was further reduced to <5% even in monolayers coated with only a low Sia-content HSM (Figure 3A, gray bars, *P*<0.05).

The clinical isolate A/SD/17/2008(H1N1) contains the oseltamivir-resistant mutation H275Y in the NA gene, as determined by cDNA sequencing (data not shown). An overall reduction of 12-44% in the number of cells infected by this strain was observed in oseltamivir-supplemented samples (Figure 3C, compare gray and black bars for each treatment). However, this effect was mostly not statistically significant, and all of the oseltamivir-supplemented samples had similar number of infected cells regardless of the mucus content (Figure 3C, compare all gray bars). Thus, the oseltamivir effect seen with this strain is likely attributed to inhibition of a secondary Sia binding site of N1 neuraminidase. In contrast, infection with A/Aichi/2/68(H3N2) strain was not affected by addition of 1 μM oseltamivir (Figure 3D, gray bars).

Taken together this suggests that NA sialidase activity is important to release the virus from the HSM layer.

### Direct evidence for cleaving of Sias by viral Neuraminidase

**Figure 4:**
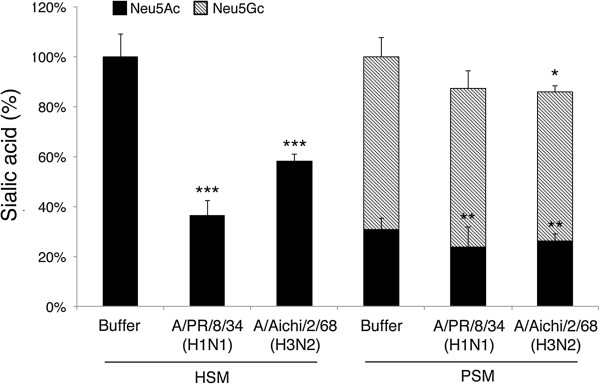
**IAV effectively cleaves sialylated HSM.** Magnetic beads-conjugated to HSM or PSM were incubated with A/PR/8/34(H1N1), A/Aichi/2/68(H3N2), or buffer at room temperature to allow cleavage of sialylated beads. After 1.5 h incubation the Sia content of the beads was analyzed by DMB-HPLC, and is expressed as percent of Sia content in buffer-incubated beads. Solid bars represent Neu5Ac content, and hatched bars represent Neu5Gc content. Both viruses reduce Neu5Ac content of HSM by 40%-60%, in contrast, only mild cleavage of PSM Sias was observed. ***P<0.001, **P<0.012 *P=0.03 values indicate the significance in difference between Sia content in the virus-treated samples and the corresponding buffer control (two-tailed T-Test, n=3).

In order to demonstrate that our viruses can cleave Sias from HSM, IAV was incubated with HSM and PSM conjugated magnetic beads. HSM and PSM were biotinylated and captured in streptavidin magnetic beads. As a control, biotinylated polyacrylamide-Galβ1-3GalNAc (T antigen) conjugated beads were also prepared. Beads were incubated with 50 μl A/PR/8/34(H1N1) (2048 HAU), A/Aichi/2/68(H3N2) (600 HAU), or DMEM-TPCK buffer for 1.5 h at room temperature to allow cleavage of sialylated beads. The beads were then extensively washed to remove both virus and cleaved (released) Sias molecules, and were then fixed with formalin. Sia content of the beads was analyzed by DMB-HPLC (Figure 4). Neu5Ac comprises 100% of the Sias in HSM samples, in contrast, Sias from PSM samples consist of ~30% Neu5Ac and 70% Neu5Gc (Additional file 1D). Both virus strains cleaved Neu5Ac from HSM, reducing the total Sia content by 40-60% compared to beads incubated with buffer alone (Figure 4, *P*<0.001). In contrast, cleavage of Sias from PSM coated beads was less effective. Both virus strains reduce Neu5Ac content by 15-23% (Figure 4, *P*<0.012), and only A/Aichi/2/68(H3N2) cleaved Neu5Gc (Figure 4, hatched bars, *P*=0.03). To our knowledge, this is the first direct demonstration that NA can cleave Sias from mucus, and we show that IAV effectively cleaves Neu5Ac from HSM but is ineffective at cleaving Sias from PSM.

### Cleaving specificity of viral neuraminidase

**Figure 5:**
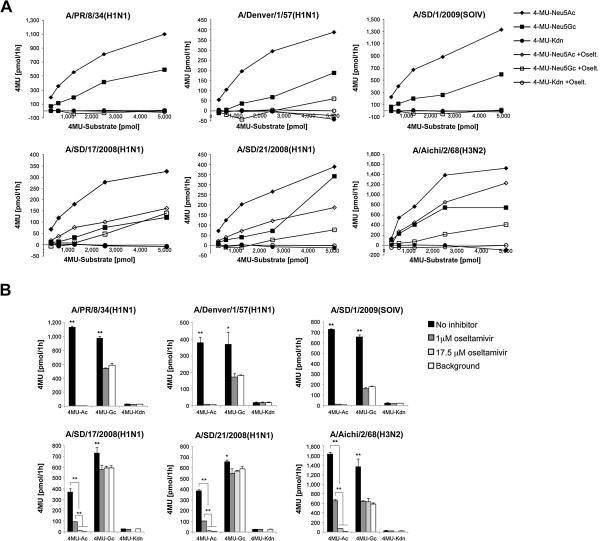
**Sia cleavage preference and susceptibility to oseltamivir inhibition. (A)** The cleaving preference of IAV NA was tested by incubation of IAV with the fluorescent reporter, 4-methyl-umbelliferyl (4MU), linked to Neu5Ac, Neu5Gc, or 2-keto-3-deoxynononic acid (Kdn) as substrate. All virus strains cleaved Neu5Ac and Neu5Gc but not Kdn. Background from spontaneous degradation of the 4MU-Sia compounds was subtracted from the results. **(B)** Virus susceptibility to oseltamivir inhibition was tested by incubation of IAV with 2.5 nmol 4MU-Neu5Ac in the presence of 0, 1 or 17.5 μM oseltamivir in triplicates. Certain strains were only partially inhibited by 1 μM oseltamivir, however, all of the strains were inhibited by 17.5 μM oseltamivir. Substrate cleavage was quantified by measuring fluorescence from the released 4MU reporter compound. *P<0.05, **P<0.01 (two-tailed T-Test, n=3).

The cleaving preference for Sia type was tested for six IAV strains using different substrates: Neu5Ac, Neu5Gc, and 2-keto-3-deoxynononic acid (Kdn), each linked to the fluorescent reporter 4-methyl-umbelliferyl (4MU, Figure 5) [30]. Virus (32-64 HAU) was diluted 10-fold in MES buffer and incubated with 4MU-Sia substrates (0-5,000 pmol) for 1 h at 37°C. The enzymatic activity of each virus NA was determined by quantifying the release of fluorescent 4MU compound. To account for spontaneous release of 4MU due to instability of the 4MU-Sia compounds (see Additional file 3), MES buffer was added instead of virus. Fluorescence in these samples was deemed background. As expected, all of the tested virus strains cleaved Neu5Ac (Figure 5A, black diamonds), and did not cleave Kdn (Figure 5A, black circles), which typically is not found in this unmodified form on mammalian tissues [31,32]. Interestingly, all six viral strains cleaved Neu5Gc as well, although to lesser extent than Neu5Ac (Figure 5A, black squares). This is surprising since the same IAV strains were ineffective at cleaving Neu5Gc from PSM (Figure 4). The viruses showed different susceptibility to inhibition by oseltamivir. Enzymatic activity of A/PR/8/34(H1N1), A/Denver/1/57(H1N1), and A/SD/1/2009(SOIV) was abolished by addition of 1 μM oseltamivir (Figure 5A and B, top panels). As expected, A/SD/17/2008(H1N1) and A/SD/21/2008(H1N1), both carrying the oseltamivir-resistant mutation H275Y in the NA gene, were only partially inhibited by 1 μM oseltamivir (Figure 5A and B, bottom panels). A/Aichi/2/68(H3N2) was also not sensitive to oseltamivir inhibition (Figure 5A and B, bottom right graphs). Importantly, all viruses were inhibited by high (17.5 μM) oseltamivir concentration (Figure 5B, *P*<0.01), thus enabling us to effectively block NA activity in order to study HA interactions with mucus (see below).

### HSM directly inhibits viral neuraminidase

**Figure 6:**
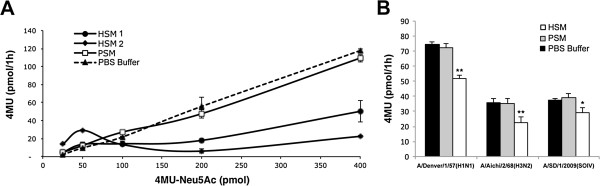
**HSM inhibits IAV cleavage of the 4MU-Neu5Ac reporter substrate. (A)** Cleavage of 4MU-Neu5Ac reporter substrate by A/Aichi/2/68(H3N2) was tested in the presence of HSM from two donors, PSM (10 nmol Sia), or PBS buffer. HSM from both donors inhibited the cleavage of 4MU-Ne5Ac. In contrast PSM did not inhibit cleavage of 4MU-Neu5Ac, similar to the buffer control. **(B)** Cleavage of 0.1 nmol 4MU-Neu5Ac by three IAV strains was tested in the presence of HSM, PSM (4.7 pmol Sia), or PBS buffer in triplicates. All virus strains were inhibited by HSM but not by PSM. Bars represent standard error, *P<0.05, **P<0.01 (two-tailed T-Test, n=3).

The ability of HSM and PSM to compete 4MU-Neu5Ac for the virus NA activity was tested by incubating virus with 4MU-Neu5Ac in the presence of mucus with 10 nmole Sia content (Figure 6). HSM but not PSM competitively inhibits the cleavage of 4MU-Neu5Ac by A/Aichi/2/68(H3N2) virus (Figure 6A). Similarly, HSM (4.7 nmol Sia content) inhibited the cleavage of 4MU-Neu5Ac (0.1 nmol) by A/Denver/1/57(H1N1), A/Aichi/2/68(H3N2), and A/SD/1/2009(SOIV). For all viruses PSM did not inhibit cleavage of 4MU-Neu5Ac (Figure 6B). Since NA affinity to 4MU-Neu5Ac compound is high [30], these findings further confirm that HSM is effectively bound by the enzymatic pocket of IAV NA.

### Direct binding of IAV to HSM on magnetic beads array

**Figure 7:**
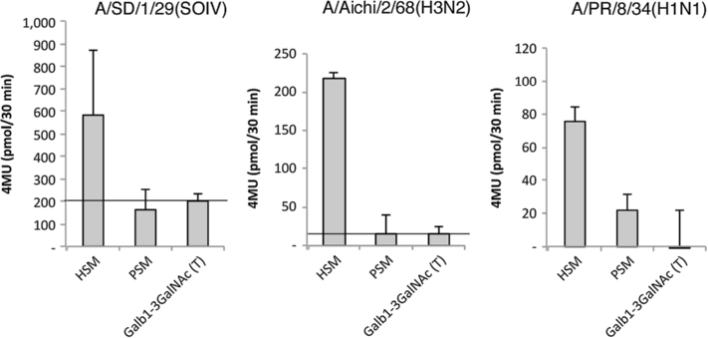
**Direct binding of IAV to HSM.** Direct binding of IAV to bead-bound HSM and PSM was tested. Virus was incubated with the mucus-coated beads in the presence of oseltamivir to inhibit NA activity. Following incubation, beads were washed to remove both non-bound virus and oseltamivir. The bound virus was quantified by measuring NA activity using 4MU-Neu5Ac-reporter method. Average of three independent experiments is shown. As control for non-specific binding, beads conjugated to non-sialylated polymer were used. Black line indicates the background. All virus strains bound to HSM, but only one strain bound to PSM.

IAV binding to sialylated mucus was tested incubating virus with HSM and PSM conjugated to magnetic beads. As control, we used magnetic beads conjugated to a non-sialylated mucus-like polyacrylamide polymer. A/PR/8/34(H1N1), A/SD/1/2009(SOIV) and A/Aichi/2/68(H3N2) viruses (32-64 HAU) were incubated with the beads for 1 h at 37°C. In order to avoid cleavage of sialylated epitope and release of the virus, 16 μM oseltamivir was added to the virus and to the wash buffer. Following incubation, the beads were washed extensively to remove both unbound virus and oseltamivir. NA regains normal activity once oseltamivir was removed (data not shown). In order to quantify the bead-bound virus, each sample was incubated with 10 nmol 4MU-Neu5Ac compound for 30 min at 37°C, in the absence of oseltamivir. The release of fluorescent 4MU compound directly correlates with the number of virions in the sample. All three tested strains bound to HSM, but only A/PR/8/34(H1N1) bound to PSM (Figure 7). In addition virus binding to magnetic beads conjugated to an array of sialylated polyacrylamide polymer standards was tested (Additional file 4). The virus-binding pattern to the standard array was in agreement with previous reports [33-36] (Additional file 4). This confirms that the glycan array method produces reliable results. Thus the balance between HA binding- and NA cleaving- of the sialylated mucus protective layer determines the ability of mucus to protect underlying cells from infection.

## Discussion

We studied the interactions between IAV and host secreted mucins. Mucins are highly sialylated secretions usually standing (or rather flowing) between the viruses and their target cells on host epithelia. They are part of a “chemical shield”, packed with defensive molecules and innate immune cells [37], and also form a mechanical clearance mechanism [8,38,39]. The small number of existing studies of the human saliva inhibitory activity on IAV infection identify several salivary molecules as potential inhibitors including surfactants, secretory IgA, histatins, defensins and MUC5B mucin [28,40]. In order to focus on the sialylated mucin aspect of the respiratory tract, we enriched the mucin component of human saliva and porcine submaxillary mucus samples by acid precipitation. Since the Sia content of the mucus samples was controlled throughout the experiments we were able to provide direct experimental evidence for the mechanism of mucus-protection during initial IAV infection.

We measured interactions of several different IAV strains with mucins *in vitro*. IAV bind to Sias on mucins (Figures 1, 2, and 7), and actively remove Sias from some mucin targets *in vitro* (Figure 4). We further show that a layer of HSM protects underlying cells from infection in Sia-dose dependent manner (Figure 3) and that this protection is augmented when viral NA (sialidase) is inhibited by oseltamivir *in vitro* (Figure 3A-B, gray bars). This inhibition was dependent on the source of mucins. Three of the IAV strains that were tested were not inhibited by PSM (Figure 3A-B, and D) but all of the tested strains were inhibited by HSM (Figure 3). Despite having a similar Neu5Ac content (1,800 and 1,500 pmol Neu5Ac, respectively), PSM (high) and HSM (low) do not have the same inhibitory effect on IAV infection. HSM (low) significantly reduced viral infection of three strains (A/PR/8/34(H1N1), A/SD/17/2008(H1N1), and A/Aichi/2/68(H3N2)), but PSM (high) only affected one of the strains (A/SD/17/2008(H1N1)) and was less efficient compared with HSM (low) (Figure 3C). Similarly, HSM competitively inhibited sialidase activity of viral NA as measured by 4MU-Sia cleavage assays, but PSM did not (Figure 6). Inhibition of NA activity with 1 μM oseltamivir reduced the number of infected cells in HSM-coated monolayers down to <10%, depending on the strain (Figure 3A-B), thus exhibiting an additive inhibitory effect. The NA of certain H1N1 strains, including the pandemic A/California/04/2009(H1N1) contains a functional secondary Sia-binding site, which can bind oseltamivir [29]. Indeed oseltamivir reduced the number of cells infected with A/SD/2009(SOIV) by ~60% even in the absence of mucus (Figure 3B); a similar trend was observed for the other two H1N1 strains (Figure 3A and C), but not for the H3N2 strain (Figure 3D). However, an additive inhibitory effect was observed only for the HSM-coated cells (Figure 3A-B, see Additional file 2 for complete statistical analysis). Thus oseltamivir impairs both the escape from sialylated mucin decoy and the binding to target cells, by pandemic IAV.

Incidentally, early studies of human saliva have reported the presence of Neu2en5Ac (N-Acetyl-2,3-dehydro-2-deoxyneuraminic Acid) which is a very potent sialidase inhibitor [41,42]. Modifications at the C-5 position of Neu2en5Ac were the first improved *in vitro* NA inhibitors [9]. However, neither Neu2en5Ac nor the modified compound has an *in vivo* inhibition activity against IAV, possibly due to their rapid clearance [9]. The presence of Neu2enAc in natural mucins might indicate that hosts also secrete their own NA-inhibitors. Alternatively, the presence of Neu2enAc could be due to activity of sialidases in saliva as this molecule also represents an intermediate product of sialidase activity.

Binding specificity of IAV strains is often studied using glycan arrays [43]. These are highly informative but lack the 3D aspect of host cells as well as the overlaying secretions. Mucins can be conceived as the “fluid glycan arrays of nature” thus utilizing mucins for probing viral functions could be immensely informative. Furthermore, due to the constant clearance of airway mucus layer, attenuating the rate of IAV penetration through the mucus layer may be sufficient to prevent infection of the underlying epithelium. Considering the additive inhibitory effect of mucins and NA inhibition, it may be possible to design NA-inhibiting drugs that will have minimal side effects. Humans and other mammals express four NA genes (NEU 1-4), which are present in the brain among other tissues [44]. It is conceivable that some of the reported neuropsychiatric adverse effects of oseltamivir and zanamavir [45,46] could be caused by cross-reaction with endogenous NA in the brain. Our results warrant further studies to better establish the function of viral NA during *in vivo* infection and to establish ways by which its role during initial infection could be perturbed. Although most studies to date have focused on the potential ability of NA inhibitors to prevent egress of viral particles from infected cells, our study confirms that these agents might also have a critical impact on viral ingress. Specifically we demonstrate that NA role during initial infection includes cleavage of sialylated mucin decoys to allow virus penetration through the mucus layer. Thus a better understanding of the NA and HA interactions within the complex molecular “ecosystem” of host mucins during initial infection also promises new avenues for drug testing and development.

## Conclusion

In this study, we show that mucins protect underlying cells from IAV infection in a sialic acid-dose dependent manner. We demonstrate direct binding to- and cleavage of- sialylated human mucins by multiple IAV strains. Our findings extend previous studies of inhibition by oseltamivir [21], providing experimental evidence for the specific molecular function of NA during initial infection. We show that NA sialidase activity is required to free virions from sialylated host mucins decoy. Thus sialylated host mucins have an important protective role against IAV infection. Sia composition, presentation and density, are critical for effective inhibition of the virus. Understanding IAV interactions with the complex molecular “ecosystem” of sialylated host mucins promises new avenues for drug testing and development.

## Methods

### Viruses and cultured cells

Three influenza strains A/PR/8/34(H1N1), A/Denver/1/57(H1N1) and A/Aichi/2/68(H3N2) were purchased from ATCC. In addition, three clinical virus isolates A/SD/21/2008(H1N1) and A/SD/17/2008(H1N1) and A/SD/1/2009(SOIV) [47,48] were also used in this study. MDCK cells were maintained in Dulbeco’s modified Eagle’s medium (DMEM, Cellgro) supplemented with 10% fetal calf serum (FCS). All viruses were propagated in MDCK cells that were transferred to DMEM medium supplemented with 0.2% BSA fraction V (EMD), 25 mM HEPES buffer (Gibco), 2 μg/ml TPCK-trypsin (Worthington Corporation), and 1% penicillin/streptomycin (“DMEM-TPCK”).

### Antibodies and lectins

Anti-Influenza A nucleoprotein monoclonal antibody (Anti-NP, MIA-NP-108) was purchased from eEnzyme, TKH2 monoclonal antibody (HB-9654) was purchased from ATCC, biotinylated *Sambucus nigra* lectin (bSNA) was purchased from Vector laboratories. Biotinylated donkey-anti-mouse IgG antibody and streptavidin conjugated horseradish peroxidase (SA-HRP) were purchased from Jackson Immunoresearch.

### Biotinylated probes and mucins

Biotinylated-polyacrylamide (PAA) glycan probes were purchased from GlycoTech. Probes used in this study were: Neu5Ac-PAA-biotin, Neu5Gc-PAA-biotin, Neu5Ac-α2-6GalNAc-PAA-biotin, Neu5Gc-α2-6GalNAc-PAA-biotin, 3’ Sialyllactose-PAA-biotin, 6’ Sialyllactose-PAA-biotin, and Galβ1-3GalNAc-PAA-biotin. Human submaxillary mucins (HSM) and porcine submaxillary mucins (PSM) were biotinylated with EZ-link-NHS-PEG4-biotin (Thermo Scientific) according to the manufacturer’s instructions. Free biotin was removed by dialysis against PBS in a Slide-A-Lyzer dialysis device (Thermo Scientific).

### Mucus preparation

Salivary mucins were produced as previously described with slight modifications [28]. Briefly, human saliva was collected from healthy human donors into tubes and placed on ice, with the approval of the UCSD Human Research Protections Program (protocol #080011). Saliva samples were centrifuged 5 min at 10,000 g, and the pellet was discarded. One percent penicillin/streptomycin was added to the clear supernatant. Mucins were precipitated by adjusting to pH 3.5 with 50 mM hydrochloric acid (HCl), stirring over night at 4°C, and collecting at 800 g for 10 min. The pellet was washed with miliQ water, resuspended in miliQ water, adjusted to pH 7.0 and boiled for 10 min to inhibit protease and glycosidase activity. Mucins were dialyzed against water in a 10,000 MWCO Slide-A-Lyzer dialysis device (Thermo Scientific), and stored in aliquots at -80°C. PSM was prepared following a published protocol [49]. The purity of the mucins was determined by analysis of monosaccharide composition using high performance anion exchange chromatography with Pulsed Amperometric Detection (HPAEC-PAD). In addition, amino acid content was determined by Gas Chromatography Mass Spectrometry (GC-MS) methods (Additional file 1) [50], both done at the UCSD Glycotechnology Core Resource.

### Sialic acid quantification by DMB-HPLC

Sia content was determined by 1,2-diamino-4,5-methylenedioxybenzene dihydrochloride–high performance liquid chromatography analysis (DMB-HPLC) according to a published protocol [51]. Samples were incubated for 1 h with 0.1 M HCl at 80°C to release Sias, and filtered through microcon-10kDa filtration device (Millipore). Free Sias were incubated for 2.5 h at 50°C in the dark with 7 mM DMB (Sigma), 0.75 M 2-mercapto-ethanol, 18 mM Na-hydrosulfite in 1.4 M acetic acid. Sias were separated on 250×4.6 mm Gemini C18 column (Phenomenex) with 7% MeOH, 8% Acetonitrile, 85% H_2_O solution at 0.9 ml/min using the ELITE Lachrom HPLC system (Hitachi). DMB-labeled Sias were detected at EX=373 EM=488.

### Virus tissue-binding assay

Snap frozen human tissues were obtained as part of the UCSD approved IRB protocol #101754. Tissues were embedded in optimal cutting media (OCT) and cut into 5 micrometer-thick sections by Leica CM1800 cryomicrotome. Tissues were air-dried and fixed for 30 min in 10% buffered formalin, blocked with an Avidin/Biotin blocking kit according to the manufacturer’s instructions (Vector Laboratories), followed by a 20 min incubation with 0.3% H_2_O_2_ in PBS, and a 10 min incubation with 1% bovine serum albumin in PBS (BSA/PBS). This standard procedure does not affect glycan presentation or virus binding to the tissue. Virus was concentrated using a microcon-10 filtration system (Millipore); approximately 600 hemagglutinating Units (HAU) were mixed 1:1 with 1% BSA/PBS and incubated on the tissues for 1.5 h at room temperature [52]. The virus was fixed after rinsing with 1:1 methanol/acetone for 15 min at -20°C. Tissue sections were then incubated with Anti-NP antibody (1:100) for 1 hour at room temperature, followed by 30 min incubation with biotinylated donkey-anti-mouse IgG antibody (1:500), and a 30 min incubation with SA-HRP (1:500). As a control for sialic acid specific binding, some tissue sections were incubated with 0.25 mM *Arthrobacter ureafaciens* sialidase in 50 mM sodium Acetate buffer for 2 hours at 37°C, or with 2 mM ice-cold sodium periodate for 30 min at 4°C prior to formalin fixation. Tissues were also stained with TKH2 antibody (1:50) over night at 4°C, followed by 30 min incubation with biotinylated-donkey-anti-mouse-IgG antibody (1:500), and 30 min with SA-HRP (1:500), or with biotinylated SNA (1:1000) 1 h at room temperature, followed by 30 min incubation with SA-HRP (1:500). Color was developed using Chromagen AEC (SK 4200, Vector laboratories) and nuclei were counterstained with Mayer Hematoxylin (Sigma-Aldrich). All antibodies and lectins were diluted in 1% BSA/PBS. Hematoxylin and Eosin (H&E), and Periodic Acid Schiff (PAS, Electron Microscopy Sciences) staining was done as previously described [25]. Microscopy slides were scanned with a NanoZoomer microscope (NanoZoomer 2.0 series, Hamamatsu, Japan).

### Infection inhibition assay

MDCK cells in 16-well Lab-Tek chamber slides (Nunc, Thermo Fisher Scientific, NY) were washed with PBS, and layered with 60 μl HSM or PSM diluted in PBS with Ca^2+^ and Mg^2+^ (Gibco) to 30, 64, or 240 pmol Sia/μl, or with 60 μl PBS with Ca^2+^ and Mg^2+^. Virus was diluted in DMDM-TPCK media to 10^9^ TCID50/ml, and aliquot into two vials, one of the vials contained 2 μM oseltamivir. TCID50 of the virus was calculated according to Spearman-Karber method. The cells were inoculated with 60 μl virus for 1 h at 37°C, washed three times with PBS, and fresh DMEM-TPCK media was added. Following 5.5 h incubation at 37°C, the cells were washed with PBS, fixed for 20 min with 3% paraformaldehyde, permeabilized for 4 min with 0.2% Triton X-100 (Sigma) in PBS, and blocked with 1% BSA/PBS. Media chamber and gasket were detached from the slides and the cells were incubated with Anti-NP antibody (1:100) for 1 hour at room temperature, followed by 30 min incubation with Alexa-488 conjugated donkey-anti-mouse IgG antibody (1:500), Both antibodies were diluted in 1% BSA/PBS. Nuclei were counterstained with Hoechst stain (Life Technologies, NY). Samples were analyzed with DeltaVision Deconvolution Microscope equipped with Coolsnap HQ camera (Applied Precision, WA). Twenty randomly selected images were taken at ×10 magnification from each sample, and the number of cells in each image was determined using Volocity image analysis software (PerkinElmer, MA). The experiment was repeated three times. Data was analyzed by 3-way ANOVA, corrected for multiple comparisons using Tukey’s HSD.

### 4MU-Sia cleavage assay

NA activity was measured using 2’-(4-methylumbelliferyl)-α-D-*N*-acetylneuraminic acid (4MU-Neu5Ac)(Sigma-Aldrich), 4MU-*N*-glycolyl-neuraminic acid (4MU-Neu5Gc) and 4MU-2-keto-3-deoxynononic acid (4MU-Kdn) (both gifts from Ken Kitajima, Nagoya University, Japan) as a substrate as previously described [30]. Briefly, 4MU-Sia compounds were used as substrates in 33 mM 2-(N-Morpholino)ethanesulphonic acid (MES, Sigma-Aldrich), 120 mM NaCl_2_, 4 mM CaCl_2_ buffer adjusted to pH 6.5 (MES/CaCl_2_/NaCl_2_ buffer). In 96-well plates, 20 μl 4MU-Sia was added to achieve a final concentration of 300-10,000 pmol sia/well. Then 30 μl virus diluted in MES/CaCl_2_/NaCl_2_ buffer was added, and incubated for 1 h at 37°C in dark. For some wells oseltamivir was added at a final concentration of 1 μM or 17.5 μM prior to incubation. HSM and PSM inhibition of 4MU-Sia cleavage was done by adding mucus at 4,500-10,000 pmol sia/well to wells containing 25-400 pmol 4MU-Neu5Ac prior to addition of virus. The reaction was stopped by adding 150 μl of 25% ethanol, 0.1M glycine pH 10.7 and measured at excitation 365 nm and emission 450 nm in a SpectraMax M3 spectrophotometer (Molecular Devices).

### Magnetic beads sialoglycan array

Sera-Mag SpeedBeads Blocked Streptavidin (Thermo-Fisher cat# 2115-2104-011150) magnetic beads were washed in PBS, and incubated with biotinylated-HSM (bHSM), biotinylated-PSM (bPSM) or biotinylated-polyacrylamide (PAA) glycan probes in 125 mM PBS pH 7.4 for 1 h at room temperature. The glycan-conjugated beads were washed with PBS and incubated with virus diluted in PBS containing 16 μM oseltamivir to 32-64 HAU for 1 h at 37°C with rotation. Beads were washed three times with oseltamivir-containing PBS, and once with PBS. NA activity of bead-bound virus was quantified by adding 100 nmol of 4MU-Neu5Ac and incubating for 30 min at 37°C. Cleavage of 4MU-Neu5Ac, quantified as described above, is directly proportional to the number of IAV bound to the beads.

### Mucus cleaving assay

Sera-Mag SpeedBeads Blocked Streptavidin (Thermo-Fisher) magnetic beads were washed in PBS, and incubated with bHSM, bPSM, or Galβ1-3GalNAc-PAA-biotin in 125 mM PBS pH 7.4 for 1 h at room temperature. The glycan-conjugated beads were washed, resuspended in 50 μl MES/CaCl_2_/NaCl_2_ buffer, and 50 μl virus (600-2048 HAU) was added. As a control, 50 μl DMEM-TPCK media was added to the beads. Beads were incubated for 1.5 h at room temperature with rotation, washed extensively with PBS, and fixed with 10% buffered formalin for 30 min. Beads were washed, 100 μl 0.1 M HCl was added and Sias were released from the beads by a 30 min incubation at 80°C. Sias were then quantified by DMB-HPLC. Galβ1-3GalNAc-PAA-biotin conjugated beads were used as control for non-specific signal.

The data sets supporting the results of this article are included within the article and its additional files.

## Competing interest

The authors declare that they have no competing interest.

## Authors’ contributions

MC designed and carried out the experiments, analyzed the data and wrote the manuscript. X-QZ isolated, characterized, subtyped the viruses and analyzed data. HPS analyzed data. H-WC subtyped the viruses. NMV interpreted the histology data. RTS designed the experiments and interoperated the data. PG designed the experiments, interpreted the data and wrote the manuscript. All authors read and approved the final manuscript.

## Supplementary Material

Additional file 1 **Characterization of mucus samples. (A)** Mucin composition in the HSM preparation is comparable to that of the human airway epithelium submucosal glands. **(B)** Amino acid composition was analyzed by gas chromatography mass spectrometry, and **(C)** monosaccharide composition was analyzed by high performance anion exchange chromatography with Pulsed Amperometric Detection (HPAEC-PAD). **(D)** The Sialic acid (Sia) content of PSM and HSM was analyzed by DMB-HPLC. Sias in HSM are linked Neu5Acα2-6GalNAc or Neu5Acα2-3Galβ1-3GalNAc [14], in PSM Sias are found as Neu5Acα2-6GalNAc or Neu5Gcα2-6GalNAc [17]. **(E)** Boiling of HSM samples for 10 min inactivates bacterial sialidase activity, which is typically found in saliva preparations. Click here for file

Additional file 2 **Complete statistical analysis for Figure** 3**.** Results of 3-way ANOVA analysis of the data presented in Figure 3, corrected for multiple comparisons using Tukey’s HSD. Click here for file

Additional file 3 **Spontaneous degradation of 4MU-Sialic acid compounds.** All three fluorescent reporter compounds, 4-methyl-umbelliferyl (4MU), linked to Neu5Ac, Neu5Gc, or 2-keto-3-deoxynononic acid (Kdn) spontaneously degrade during 1 h incubation at 37°C. Notably 4MU-Neu5Gc is the least stable compound. The fluorescent resulting from spontaneous degradation of the reporter compounds was accounted as background and was either subtracted from the results (Figure 5A) or presented as background (Figure 5B). Click here for file

Additional file 4 **IAV binding to bead glycan array.** Binding specificity of IAV to sialylated glycoconjugates and mucus was tested on bead-glycan array. Virus binding to the array was quantified by measuring NA activity using 4MU-Neu5Ac-reporter method. Average of three independent experiments, each done in triplicates, is shown. Black line indicates the background. All virus strains bound to HSM. **(A)** A/PR/8/34(H1N1) showed a preference for Siaα2-3Lactose over Siaα2-6Lactose, did not bind to Neu5Gc containing structures, and showed weak binding to PSM. **(B)** The pandemic strain A/SD/1/2009(SOIV) exhibited a broad binding specificity, but did not bind PSM. **(C)** A/Aichi/2/68(H3N2) showed preference for Sia in α2-6 linkages (Siaα2-6Lactose and Sialyl Tn), had weak binding to Neu5Gc containing structures (Neu5Gc and Neu5Gc-SialylTn), and did not bind to PSM. Click here for file
