# Supplementary figures and images for: Influenza A penetrates host mucus by cleaving sialic acids with neuraminidase
Source: Virol J. 2013 Nov 22;10:321. doi: 10.1186/1743-422X-10-321 (PMC3842836; doi:10.1186/1743-422X-10-321)

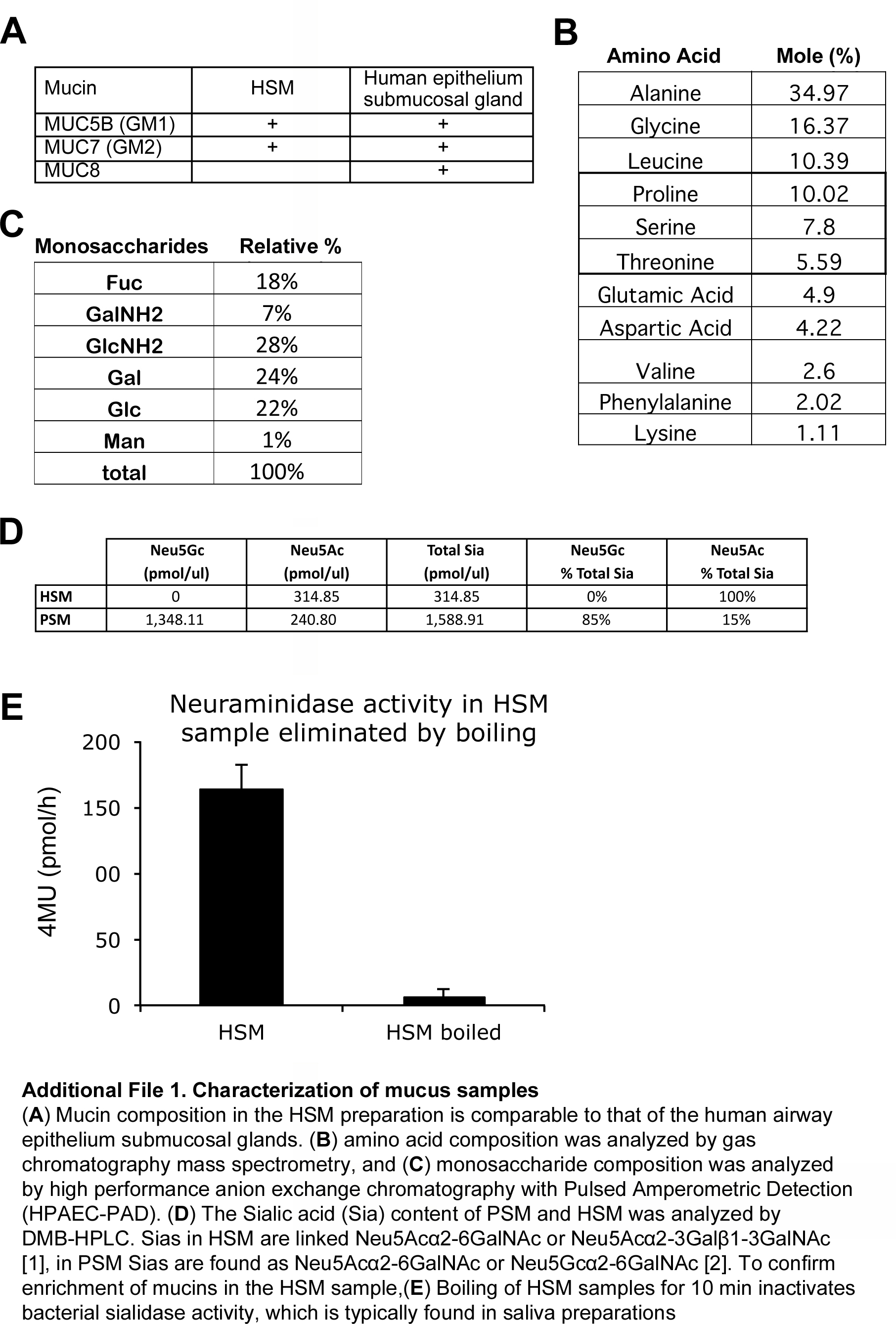

Supplement: Additional file 1 — Characterization of mucus samples. (A) Mucin composition in the HSM preparation is comparable to that of the human airway epithelium submucosal glands. (B) Amino acid composition was analyzed by gas chromatography mass spectrometry, and (C) monosaccharide composition was analyzed by high performance anion exchange chromatography with Pulsed Amperometric Detection (HPAEC-PAD). (D) The Sialic acid (Sia) content of PSM and HSM was analyzed by DMB-HPLC. Sias in HSM are linked Neu5Acα2-6GalNAc or Neu5Acα2-3Galβ1-3GalNAc [14], in PSM Sias are found as Neu5Acα2-6GalNAc or Neu5Gcα2-6GalNAc [17]. (E) Boiling of HSM samples for 10 min inactivates bacterial sialidase activity, which is typically found in saliva preparations. [file 1743-422X-10-321-S1.jpeg]

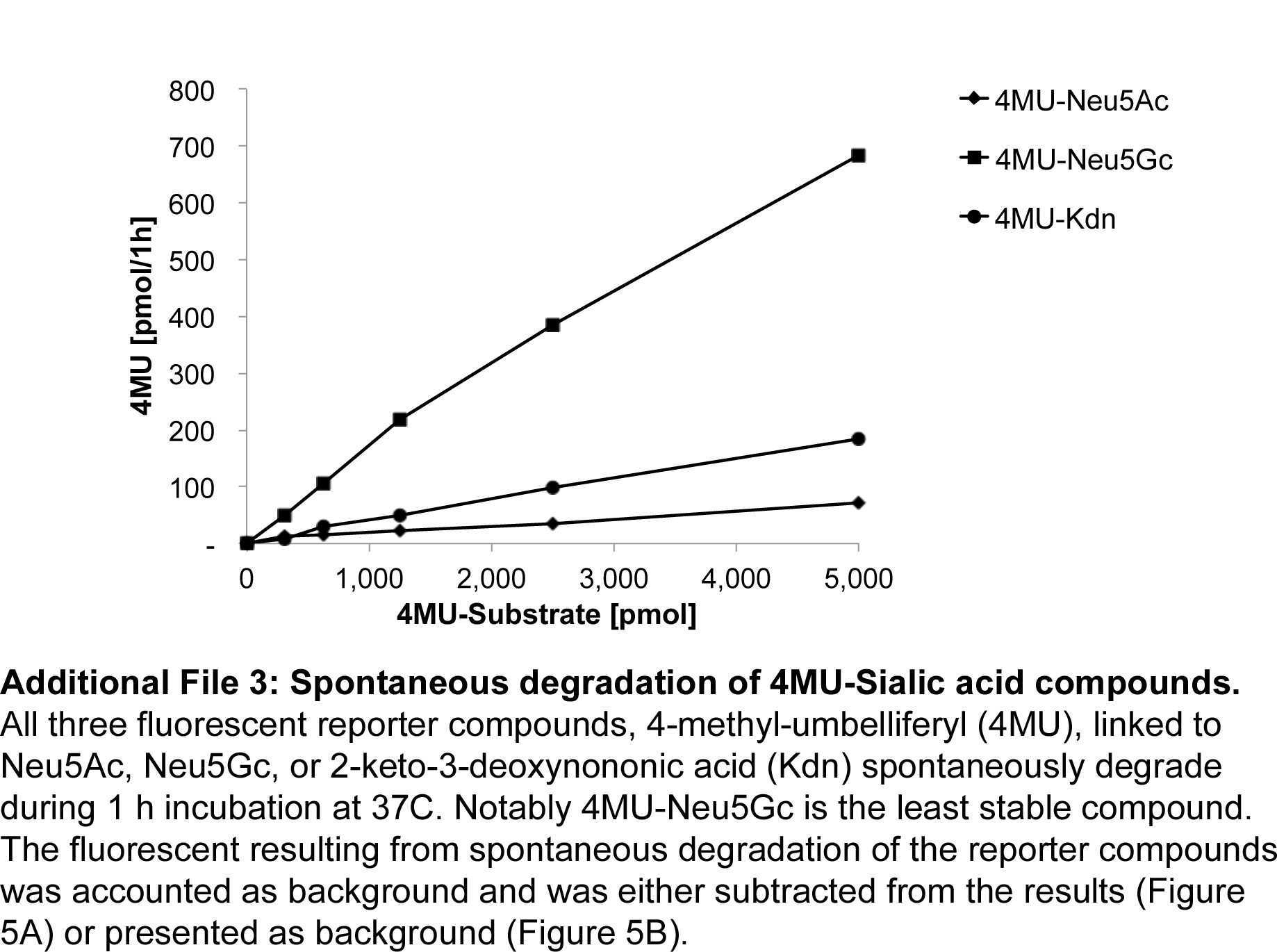

Supplement: Additional file 3 — Spontaneous degradation of 4MU-Sialic acid compounds. All three fluorescent reporter compounds, 4-methyl-umbelliferyl (4MU), linked to Neu5Ac, Neu5Gc, or 2-keto-3-deoxynononic acid (Kdn) spontaneously degrade during 1 h incubation at 37°C. Notably 4MU-Neu5Gc is the least stable compound. The fluorescent resulting from spontaneous degradation of the reporter compounds was accounted as background and was either subtracted from the results (Figure 5A) or presented as background (Figure 5B). [file 1743-422X-10-321-S3.jpeg]

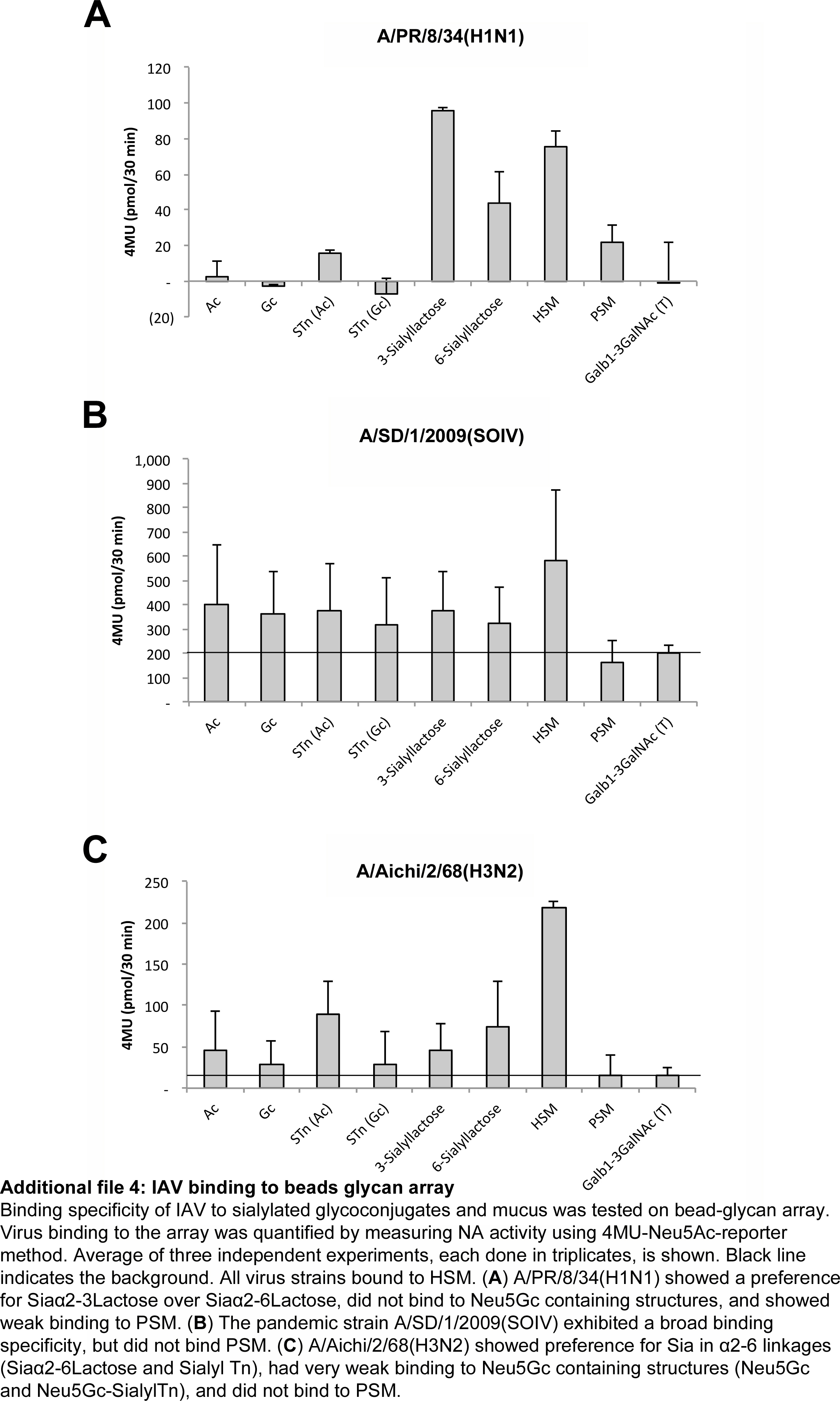

Supplement: Additional file 4 — IAV binding to bead glycan array. Binding specificity of IAV to sialylated glycoconjugates and mucus was tested on bead-glycan array. Virus binding to the array was quantified by measuring NA activity using 4MU-Neu5Ac-reporter method. Average of three independent experiments, each done in triplicates, is shown. Black line indicates the background. All virus strains bound to HSM. (A) A/PR/8/34(H1N1) showed a preference for Siaα2-3Lactose over Siaα2-6Lactose, did not bind to Neu5Gc containing structures, and showed weak binding to PSM. (B) The pandemic strain A/SD/1/2009(SOIV) exhibited a broad binding specificity, but did not bind PSM. (C) A/Aichi/2/68(H3N2) showed preference for Sia in α2-6 linkages (Siaα2-6Lactose and Sialyl Tn), had weak binding to Neu5Gc containing structures (Neu5Gc and Neu5Gc-SialylTn), and did not bind to PSM. [file 1743-422X-10-321-S4.jpeg]
