# Supplementary material for: Influenza A penetrates host mucus by cleaving sialic acids with neuraminidase
Source: Virol J. 2013 Nov 22;10:321. doi: 10.1186/1743-422X-10-321 (PMC3842836; doi:10.1186/1743-422X-10-321)
Supplement: Additional file 2 — Complete statistical analysis for Figure 3. Results of 3-way ANOVA analysis of the data presented in Figure 3, corrected for multiple comparisons using Tukey’s HSD. [file 1743-422X-10-321-S2.pdf]

Additional file 2: Results of 3-way ANOVA analysis of the data in Figure 3, corrected for multiple comparisons using Tukey's HSD.

Virus	A/PR/8/34(H1N1)		A/SD/1/2009(SOIV)		A/SD/17/2008(H1N1)		A/Aichi/2/68(H3N2)	
**PAIRWISE COMPARISONS**	**t**	**P**	**t**	**P**	**t**	**P**	**t**	**P**
[PSM_0_No]vs[PSM_0_Yes]	t(4)=2.62	p< 0.0589	t(4)=6.29	p< 0.0033	t(4)=2.47	p< 0.0689	t(4)=0.41	p< 0.7042
[PSM_0_No]vs[PSM_1500_No]	t(4)=0.21	p< 0.8468	t(4)=0.42	p< 0.6951	t(4)=3.45	p< 0.0261	t(4)=0.22	p< 0.8396
[PSM_0_No]vs[PSM_1500_Yes]	t(4)=6.08	p< 0.0037	t(4)=26.71	p< 0.0001	t(4)=11.94	p< 0.0003	t(4)=0.30	p< 0.7798
[PSM_0_No]vs[PSM_3200_No]	t(4)=0.04	p< 0.9678	t(4)=1.45	p< 0.2196	t(4)=3.83	p< 0.0186	t(4)=1.64	p< 0.1755
[PSM_0_No]vs[PSM_3200_Yes]	t(4)=10.30	p< 0.0005	t(4)=15.37	p< 0.0001	t(4)=61.00	p< 0.0001	t(4)=0.32	p< 0.7637
[PSM_0_No]vs[PSM_12000_No]	t(4)=0.02	p< 0.9861	t(4)=2.55	p< 0.0636	t(4)=5.53	p< 0.0052	t(4)=0.74	p< 0.5000
[PSM_0_No]vs[PSM_12000_Yes]	t(4)=4.34	p< 0.0123	t(4)=16.80	p< 0.0001	t(4)=19.82	p< 0.0001	t(4)=0.07	p< 0.9511
[PSM_0_No]vs[HSM_0_No]	t(4)=0.00	p< 1.0000	t(4)=0.00	p< 1.0000	t(4)=0.00	p< 1.0000	t(4)=0.00	p< 1.0000
[PSM_0_No]vs[HSM_0_Yes]	t(4)=2.62	p< 0.0589	t(4)=6.29	p< 0.0033	t(4)=2.47	p< 0.0689	t(4)=0.41	p< 0.7042
[PSM_0_No]vs[HSM_1500_No]	t(4)=10.45	p< 0.0005	t(4)=1.97	p< 0.1196	t(4)=6.84	p< 0.0024	t(4)=2.85	p< 0.0462
[PSM_0_No]vs[HSM_1500_Yes]	t(4)=39.29	p< 0.0001	t(4)=22.25	p< 0.0001	t(4)=4.07	p< 0.0152	t(4)=15.13	p< 0.0001
[PSM_0_No]vs[HSM_3200_No]	t(4)=18.31	p< 0.0001	t(4)=3.47	p< 0.0256	t(4)=3.91	p< 0.0173	t(4)=8.13	p< 0.0012
[PSM_0_No]vs[HSM_3200_Yes]	t(4)=295.00	p< 0.0001	t(4)=108.48	p< 0.0001	t(4)=6.49	p< 0.0029	t(4)=15.30	p< 0.0001
[PSM_0_No]vs[HSM_12000_No]	t(4)=50.47	p< 0.0001	t(4)=11.43	p< 0.0003	t(4)=3.88	p< 0.0179	t(4)=17.05	p< 0.0001
[PSM_0_No]vs[HSM_12000_Yes]	t(4)=169.74	p< 0.0001	t(4)=108.10	p< 0.0001	t(4)=7.53	p< 0.0017	t(4)=138.50	p< 0.0001
[PSM_0_Yes]vs[PSM_1500_No]	t(4)=1.53	p< 0.2019	t(4)=3.34	p< 0.0288	t(4)=1.30	p< 0.2647	t(4)=0.46	p< 0.6714
[PSM_0_Yes]vs[PSM_1500_Yes]	t(4)=0.17	p< 0.8734	t(4)=0.38	p< 0.7225	t(4)=0.85	p< 0.4434	t(4)=0.10	p< 0.9247
[PSM_0_Yes]vs[PSM_3200_No]	t(4)=1.45	p< 0.2204	t(4)=4.80	p< 0.0086	t(4)=0.40	p< 0.7085	t(4)=1.16	p< 0.3124
[PSM_0_Yes]vs[PSM_3200_Yes]	t(4)=0.12	p< 0.9099	t(4)=1.18	p< 0.3018	t(4)=0.60	p< 0.5782	t(4)=0.09	p< 0.9353
[PSM_0_Yes]vs[PSM_12000_No]	t(4)=1.32	p< 0.2560	t(4)=2.36	p< 0.0776	t(4)=0.72	p< 0.5100	t(4)=0.80	p< 0.4698
[PSM_0_Yes]vs[PSM_12000_Yes]	t(4)=0.74	p< 0.4977	t(4)=1.61	p< 0.1828	t(4)=0.80	p< 0.4706	t(4)=0.17	p< 0.8709
[PSM_0_Yes]vs[HSM_0_No]	t(4)=2.62	p< 0.0589	t(4)=6.29	p< 0.0033	t(4)=2.47	p< 0.0689	t(4)=0.41	p< 0.7042
[PSM_0_Yes]vs[HSM_0_Yes]	t(4)=0.00	p< 1.0000	t(4)=0.00	p< 1.0000	t(4)=0.00	p< 1.0000	t(4)=0.00	p< 1.0000
[PSM_0_Yes]vs[HSM_1500_No]	t(4)=1.98	p< 0.1184	t(4)=2.61	p< 0.0591	t(4)=0.75	p< 0.4954	t(4)=1.43	p< 0.2266
[PSM_0_Yes]vs[HSM_1500_Yes]	t(4)=5.82	p< 0.0043	t(4)=2.99	p< 0.0404	t(4)=1.21	p< 0.2932	t(4)=3.17	p< 0.0338
[PSM_0_Yes]vs[HSM_3200_No]	t(4)=3.37	p< 0.0281	t(4)=1.06	p< 0.3475	t(4)=0.09	p< 0.9354	t(4)=2.88	p< 0.0448
[PSM_0_Yes]vs[HSM_3200_Yes]	t(4)=6.57	p< 0.0028	t(4)=3.95	p< 0.0169	t(4)=1.95	p< 0.1230	t(4)=3.92	p< 0.0173
[PSM_0_Yes]vs[HSM_12000_No]	t(4)=6.05	p< 0.0038	t(4)=1.83	p< 0.1415	t(4)=1.44	p< 0.2242	t(4)=4.59	p< 0.0101
[PSM_0_Yes]vs[HSM_12000_Yes]	t(4)=6.54	p< 0.0028	t(4)=3.91	p< 0.0174	t(4)=2.67	p< 0.0559	t(4)=4.97	p< 0.0077
[PSM_1500_No]vs[PSM_1500_Yes]	t(4)=1.82	p< 0.1424	t(4)=3.97	p< 0.0166	t(4)=5.25	p< 0.0063	t(4)=0.36	p< 0.7387
[PSM_1500_No]vs[PSM_3200_No]	t(4)=0.18	p< 0.8660	t(4)=0.55	p< 0.6129	t(4)=1.47	p< 0.2153	t(4)=1.26	p< 0.2753
[PSM_1500_No]vs[PSM_3200_Yes]	t(4)=1.83	p< 0.1411	t(4)=4.35	p< 0.0121	t(4)=6.04	p< 0.0038	t(4)=0.38	p< 0.7240
[PSM_1500_No]vs[PSM_12000_No]	t(4)=0.16	p< 0.8819	t(4)=1.65	p< 0.1739	t(4)=1.35	p< 0.2471	t(4)=0.61	p< 0.5772
[PSM_1500_No]vs[PSM_12000_Yes]	t(4)=2.11	p< 0.1021	t(4)=4.60	p< 0.0100	t(4)=5.99	p< 0.0039	t(4)=0.11	p< 0.9147
[PSM_1500_No]vs[HSM_0_No]	t(4)=0.21	p< 0.8468	t(4)=0.42	p< 0.6951	t(4)=3.45	p< 0.0261	t(4)=0.22	p< 0.8396
[PSM_1500_No]vs[HSM_0_Yes]	t(4)=1.53	p< 0.2019	t(4)=3.34	p< 0.0288	t(4)=1.30	p< 0.2647	t(4)=0.46	p< 0.6714
[PSM_1500_No]vs[HSM_1500_No]	t(4)=2.98	p< 0.0408	t(4)=1.40	p< 0.2342	t(4)=3.78	p< 0.0195	t(4)=2.68	p< 0.0552
[PSM_1500_No]vs[HSM_1500_Yes]	t(4)=5.31	p< 0.0060	t(4)=5.40	p< 0.0057	t(4)=2.86	p< 0.0458	t(4)=8.64	p< 0.0010
[PSM_1500_No]vs[HSM_3200_No]	t(4)=3.84	p< 0.0185	t(4)=2.34	p< 0.0796	t(4)=1.84	p< 0.1399	t(4)=6.41	p< 0.0030
[PSM_1500_No]vs[HSM_3200_Yes]	t(4)=5.73	p< 0.0046	t(4)=5.91	p< 0.0041	t(4)=4.62	p< 0.0098	t(4)=9.83	p< 0.0006
[PSM_1500_No]vs[HSM_12000_No]	t(4)=5.44	p< 0.0055	t(4)=4.66	p< 0.0096	t(4)=2.87	p< 0.0454	t(4)=11.20	p< 0.0004
[PSM_1500_No]vs[HSM_12000_Yes]	t(4)=5.71	p< 0.0047	t(4)=5.89	p< 0.0041	t(4)=5.64	p< 0.0049	t(4)=15.08	p< 0.0001
[PSM_1500_Yes]vs[PSM_3200_No]	t(4)=1.80	p< 0.1458	t(4)=6.05	p< 0.0038	t(4)=2.24	p< 0.0887	t(4)=1.11	p< 0.3284
[PSM_1500_Yes]vs[PSM_3200_Yes]	t(4)=0.12	p< 0.9125	t(4)=1.67	p< 0.1693	t(4)=0.97	p< 0.3860	t(4)=0.02	p< 0.9886
[PSM_1500_Yes]vs[PSM_12000_No]	t(4)=1.59	p< 0.1862	t(4)=3.47	p< 0.0256	t(4)=3.92	p< 0.0173	t(4)=0.73	p< 0.5047
[PSM_1500_Yes]vs[PSM_12000_Yes]	t(4)=0.82	p< 0.4566	t(4)=2.57	p< 0.0619	t(4)=0.23	p< 0.8278	t(4)=0.10	p< 0.9250
[PSM_1500_Yes]vs[HSM_0_No]	t(4)=6.08	p< 0.0037	t(4)=26.71	p< 0.0001	t(4)=11.94	p< 0.0003	t(4)=0.30	p< 0.7798
[PSM_1500_Yes]vs[HSM_0_Yes]	t(4)=0.17	p< 0.8734	t(4)=0.38	p< 0.7225	t(4)=0.85	p< 0.4434	t(4)=0.10	p< 0.9247
[PSM_1500_Yes]vs[HSM_1500_No]	t(4)=3.07	p< 0.0375	t(4)=3.71	p< 0.0207	t(4)=0.09	p< 0.9324	t(4)=1.62	p< 0.1795
[PSM_1500_Yes]vs[HSM_1500_Yes]	t(4)=11.30	p< 0.0003	t(4)=5.76	p< 0.0045	t(4)=0.80	p< 0.4687	t(4)=3.61	p< 0.0225
[PSM_1500_Yes]vs[HSM_3200_No]	t(4)=5.89	p< 0.0041	t(4)=1.61	p< 0.1826	t(4)=1.47	p< 0.2147	t(4)=3.25	p< 0.0313
[PSM_1500_Yes]vs[HSM_3200_Yes]	t(4)=13.82	p< 0.0002	t(4)=13.36	p< 0.0002	t(4)=1.93	p< 0.1251	t(4)=4.41	p< 0.0116
[PSM_1500_Yes]vs[HSM_12000_No]	t(4)=12.08	p< 0.0003	t(4)=2.39	p< 0.0748	t(4)=1.11	p< 0.3311	t(4)=5.14	p< 0.0068
[PSM_1500_Yes]vs[HSM_12000_Yes]	t(4)=13.69	p< 0.0002	t(4)=13.23	p< 0.0002	t(4)=3.02	p< 0.0391	t(4)=5.61	p< 0.0049
[PSM_3200_No]vs[PSM_3200_Yes]	t(4)=1.82	p< 0.1432	t(4)=6.42	p< 0.0030	t(4)=2.00	p< 0.1158	t(4)=1.13	p< 0.3201
[PSM_3200_No]vs[PSM_12000_No]	t(4)=0.01	p< 0.9895	t(4)=2.70	p< 0.0539	t(4)=0.50	p< 0.6464	t(4)=0.24	p< 0.8218
[PSM_3200_No]vs[PSM_12000_Yes]	t(4)=2.11	p< 0.1024	t(4)=6.75	p< 0.0025	t(4)=2.28	p< 0.0844	t(4)=0.64	p< 0.5597
[PSM_3200_No]vs[HSM_0_No]	t(4)=0.04	p< 0.9678	t(4)=1.45	p< 0.2196	t(4)=3.83	p< 0.0186	t(4)=1.64	p< 0.1755
[PSM_3200_No]vs[HSM_0_Yes]	t(4)=1.45	p< 0.2204	t(4)=4.80	p< 0.0086	t(4)=0.40	p< 0.7085	t(4)=1.16	p< 0.3124
[PSM_3200_No]vs[HSM_1500_No]	t(4)=3.12	p< 0.0357	t(4)=2.37	p< 0.0765	t(4)=1.80	p< 0.1463	t(4)=3.28	p< 0.0306

Virus	A/PR/8/34(H1N1)		A/SD/1/2009(SOIV)		A/SD/17/2008(H1N1)		A/Aichi/2/68(H3N2)	
**PAIRWISE COMPARISONS**	**t**	**P**	**t**	**P**	**t**	**P**	**t**	**P**
[PSM_3200_No]vs[HSM_1500_Yes]	t(4)=5.80	p< 0.0044	t(4)=7.80	p< 0.0015	t(4)=1.89	p< 0.1318	t(4)=7.57	p< 0.0016
[PSM_3200_No]vs[HSM_3200_No]	t(4)=4.10	p< 0.0148	t(4)=3.42	p< 0.0268	t(4)=0.44	p< 0.6828	t(4)=6.32	p< 0.0032
[PSM_3200_No]vs[HSM_3200_Yes]	t(4)=6.30	p< 0.0033	t(4)=8.63	p< 0.0010	t(4)=3.14	p< 0.0348	t(4)=8.60	p< 0.0010
[PSM_3200_No]vs[HSM_12000_No]	t(4)=5.96	p< 0.0040	t(4)=6.61	p< 0.0027	t(4)=2.05	p< 0.1103	t(4)=9.66	p< 0.0006
[PSM_3200_No]vs[HSM_12000_Yes]	t(4)=6.27	p< 0.0033	t(4)=8.61	p< 0.0010	t(4)=4.08	p< 0.0150	t(4)=11.31	p< 0.0003
[PSM_3200_Yes]vs[PSM_12000_No]	t(4)=1.60	p< 0.1858	t(4)=4.01	p< 0.0159	t(4)=4.34	p< 0.0123	t(4)=0.75	p< 0.4955
[PSM_3200_Yes]vs[PSM_12000_Yes]	t(4)=0.97	p< 0.3873	t(4)=0.67	p< 0.5378	t(4)=1.17	p< 0.3081	t(4)=0.11	p< 0.9166
[PSM_3200_Yes]vs[HSM_0_No]	t(4)=10.30	p< 0.0005	t(4)=15.37	p< 0.0001	t(4)=61.00	p< 0.0001	t(4)=0.32	p< 0.7637
[PSM_3200_Yes]vs[HSM_0_Yes]	t(4)=0.12	p< 0.9099	t(4)=1.18	p< 0.3018	t(4)=0.60	p< 0.5782	t(4)=0.09	p< 0.9353
[PSM_3200_Yes]vs[HSM_1500_No]	t(4)=3.88	p< 0.0179	t(4)=4.23	p< 0.0134	t(4)=0.47	p< 0.6646	t(4)=1.61	p< 0.1821
[PSM_3200_Yes]vs[HSM_1500_Yes]	t(4)=16.93	p< 0.0001	t(4)=2.95	p< 0.0421	t(4)=1.10	p< 0.3349	t(4)=3.61	p< 0.0226
[PSM_3200_Yes]vs[HSM_3200_No]	t(4)=7.98	p< 0.0013	t(4)=2.20	p< 0.0929	t(4)=1.16	p< 0.3100	t(4)=3.25	p< 0.0315
[PSM_3200_Yes]vs[HSM_3200_Yes]	t(4)=24.06	p< 0.0001	t(4)=5.25	p< 0.0063	t(4)=2.42	p< 0.0725	t(4)=4.41	p< 0.0116
[PSM_3200_Yes]vs[HSM_12000_No]	t(4)=18.93	p< 0.0001	t(4)=1.07	p< 0.3436	t(4)=1.36	p< 0.2456	t(4)=5.14	p< 0.0068
[PSM_3200_Yes]vs[HSM_12000_Yes]	t(4)=23.63	p< 0.0001	t(4)=5.17	p< 0.0066	t(4)=3.56	p< 0.0235	t(4)=5.62	p< 0.0049
[PSM_12000_No]vs[PSM_12000_Yes]	t(4)=1.90	p< 0.1303	t(4)=4.42	p< 0.0115	t(4)=4.45	p< 0.0112	t(4)=0.44	p< 0.6851
[PSM_12000_No]vs[HSM_0_No]	t(4)=0.02	p< 0.9861	t(4)=2.55	p< 0.0636	t(4)=5.53	p< 0.0052	t(4)=0.74	p< 0.5000
[PSM_12000_No]vs[HSM_0_Yes]	t(4)=1.32	p< 0.2560	t(4)=2.36	p< 0.0776	t(4)=0.72	p< 0.5100	t(4)=0.80	p< 0.4698
[PSM_12000_No]vs[HSM_1500_No]	t(4)=2.74	p< 0.0519	t(4)=0.31	p< 0.7693	t(4)=2.77	p< 0.0504	t(4)=2.43	p< 0.0718
[PSM_12000_No]vs[HSM_1500_Yes]	t(4)=5.05	p< 0.0072	t(4)=5.74	p< 0.0046	t(4)=2.31	p< 0.0819	t(4)=4.69	p< 0.0094
[PSM_12000_No]vs[HSM_3200_No]	t(4)=3.59	p< 0.0230	t(4)=1.03	p< 0.3626	t(4)=0.96	p< 0.3898	t(4)=4.24	p< 0.0132
[PSM_12000_No]vs[HSM_3200_Yes]	t(4)=5.46	p< 0.0055	t(4)=6.80	p< 0.0024	t(4)=3.91	p< 0.0174	t(4)=5.48	p< 0.0054
[PSM_12000_No]vs[HSM_12000_No]	t(4)=5.17	p< 0.0066	t(4)=4.36	p< 0.0121	t(4)=2.40	p< 0.0745	t(4)=6.22	p< 0.0034
[PSM_12000_No]vs[HSM_12000_Yes]	t(4)=5.44	p< 0.0055	t(4)=6.76	p< 0.0025	t(4)=4.94	p< 0.0078	t(4)=6.77	p< 0.0025
[PSM_12000_Yes]vs[HSM_0_No]	t(4)=4.34	p< 0.0123	t(4)=16.80	p< 0.0001	t(4)=19.82	p< 0.0001	t(4)=0.07	p< 0.9511
[PSM_12000_Yes]vs[HSM_0_Yes]	t(4)=0.74	p< 0.4977	t(4)=1.61	p< 0.1828	t(4)=0.80	p< 0.4706	t(4)=0.17	p< 0.8709
[PSM_12000_Yes]vs[HSM_1500_No]	t(4)=1.29	p< 0.2679	t(4)=4.62	p< 0.0099	t(4)=0.05	p< 0.9630	t(4)=1.26	p< 0.2760
[PSM_12000_Yes]vs[HSM_1500_Yes]	t(4)=5.83	p< 0.0043	t(4)=2.27	p< 0.0853	t(4)=0.89	p< 0.4236	t(4)=2.37	p< 0.0770
[PSM_12000_Yes]vs[HSM_3200_No]	t(4)=2.90	p< 0.0442	t(4)=2.54	p< 0.0640	t(4)=1.45	p< 0.2218	t(4)=2.23	p< 0.0899
[PSM_12000_Yes]vs[HSM_3200_Yes]	t(4)=6.78	p< 0.0025	t(4)=4.45	p< 0.0113	t(4)=2.11	p< 0.1021	t(4)=2.89	p< 0.0445
[PSM_12000_Yes]vs[HSM_12000_No]	t(4)=6.13	p< 0.0036	t(4)=0.56	p< 0.6045	t(4)=1.18	p< 0.3016	t(4)=3.36	p< 0.0283
[PSM_12000_Yes]vs[HSM_12000_Yes]	t(4)=6.74	p< 0.0025	t(4)=4.38	p< 0.0119	t(4)=3.24	p< 0.0317	t(4)=3.55	p< 0.0239
[HSM_0_No]vs[HSM_0_Yes]	t(4)=2.62	p< 0.0589	t(4)=6.29	p< 0.0033	t(4)=2.47	p< 0.0689	t(4)=0.41	p< 0.7042
[HSM_0_No]vs[HSM_1500_No]	t(4)=10.45	p< 0.0005	t(4)=1.97	p< 0.1196	t(4)=6.84	p< 0.0024	t(4)=2.85	p< 0.0462
[HSM_0_No]vs[HSM_1500_Yes]	t(4)=39.29	p< 0.0001	t(4)=22.25	p< 0.0001	t(4)=4.07	p< 0.0152	t(4)=15.13	p< 0.0001
[HSM_0_No]vs[HSM_3200_No]	t(4)=18.31	p< 0.0001	t(4)=3.47	p< 0.0256	t(4)=3.91	p< 0.0173	t(4)=8.13	p< 0.0012
[HSM_0_No]vs[HSM_3200_Yes]	t(4)=295.00	p< 0.0001	t(4)=108.48	p< 0.0001	t(4)=6.49	p< 0.0029	t(4)=15.30	p< 0.0001
[HSM_0_No]vs[HSM_12000_No]	t(4)=50.47	p< 0.0001	t(4)=11.43	p< 0.0003	t(4)=3.88	p< 0.0179	t(4)=17.05	p< 0.0001
[HSM_0_No]vs[HSM_12000_Yes]	t(4)=169.74	p< 0.0001	t(4)=108.10	p< 0.0001	t(4)=7.53	p< 0.0017	t(4)=138.50	p< 0.0001
[HSM_0_Yes]vs[HSM_1500_No]	t(4)=1.98	p< 0.1184	t(4)=2.61	p< 0.0591	t(4)=0.75	p< 0.4954	t(4)=1.43	p< 0.2266
[HSM_0_Yes]vs[HSM_1500_Yes]	t(4)=5.82	p< 0.0043	t(4)=2.99	p< 0.0404	t(4)=1.21	p< 0.2932	t(4)=3.17	p< 0.0338
[HSM_0_Yes]vs[HSM_3200_No]	t(4)=3.37	p< 0.0281	t(4)=1.06	p< 0.3475	t(4)=0.09	p< 0.9354	t(4)=2.88	p< 0.0448
[HSM_0_Yes]vs[HSM_3200_Yes]	t(4)=6.57	p< 0.0028	t(4)=3.95	p< 0.0169	t(4)=1.95	p< 0.1230	t(4)=3.92	p< 0.0173
[HSM_0_Yes]vs[HSM_12000_No]	t(4)=6.05	p< 0.0038	t(4)=1.83	p< 0.1415	t(4)=1.44	p< 0.2242	t(4)=4.59	p< 0.0101
[HSM_0_Yes]vs[HSM_12000_Yes]	t(4)=6.54	p< 0.0028	t(4)=3.91	p< 0.0174	t(4)=2.67	p< 0.0559	t(4)=4.97	p< 0.0077
[HSM_1500_No]vs[HSM_1500_Yes]	t(4)=7.42	p< 0.0018	t(4)=5.87	p< 0.0042	t(4)=0.79	p< 0.4712	t(4)=1.79	p< 0.1476
[HSM_1500_No]vs[HSM_3200_No]	t(4)=2.41	p< 0.0737	t(4)=1.29	p< 0.2670	t(4)=1.20	p< 0.2949	t(4)=1.50	p< 0.2069
[HSM_1500_No]vs[HSM_3200_Yes]	t(4)=9.55	p< 0.0007	t(4)=6.86	p< 0.0024	t(4)=1.80	p< 0.1469	t(4)=2.74	p< 0.0519
[HSM_1500_No]vs[HSM_12000_No]	t(4)=8.06	p< 0.0013	t(4)=4.56	p< 0.0104	t(4)=1.09	p< 0.3358	t(4)=3.59	p< 0.0230
[HSM_1500_No]vs[HSM_12000_Yes]	t(4)=9.44	p< 0.0007	t(4)=6.83	p< 0.0024	t(4)=2.78	p< 0.0496	t(4)=4.07	p< 0.0152
[HSM_1500_Yes]vs[HSM_3200_No]	t(4)=5.98	p< 0.0039	t(4)=3.64	p< 0.0221	t(4)=1.54	p< 0.1995	t(4)=0.23	p< 0.8272
[HSM_1500_Yes]vs[HSM_3200_Yes]	t(4)=2.83	p< 0.0474	t(4)=1.63	p< 0.1789	t(4)=0.55	p< 0.6113	t(4)=2.14	p< 0.0987
[HSM_1500_Yes]vs[HSM_12000_No]	t(4)=0.67	p< 0.5391	t(4)=1.12	p< 0.3268	t(4)=0.33	p< 0.7576	t(4)=3.94	p< 0.0170
[HSM_1500_Yes]vs[HSM_12000_Yes]	t(4)=2.63	p< 0.0579	t(4)=1.55	p< 0.1970	t(4)=1.27	p< 0.2740	t(4)=6.96	p< 0.0022
[HSM_3200_No]vs[HSM_3200_Yes]	t(4)=8.93	p< 0.0009	t(4)=4.36	p< 0.0121	t(4)=2.63	p< 0.0585	t(4)=1.77	p< 0.1514
[HSM_3200_No]vs[HSM_12000_No]	t(4)=6.82	p< 0.0024	t(4)=2.68	p< 0.0553	t(4)=1.74	p< 0.1575	t(4)=3.10	p< 0.0364
[HSM_3200_No]vs[HSM_12000_Yes]	t(4)=8.76	p< 0.0009	t(4)=4.33	p< 0.0123	t(4)=3.53	p< 0.0242	t(4)=4.16	p< 0.0142
[HSM_3200_Yes]vs[HSM_12000_No]	t(4)=2.47	p< 0.0686	t(4)=2.22	p< 0.0906	t(4)=0.12	p< 0.9083	t(4)=1.70	p< 0.1648
[HSM_3200_Yes]vs[HSM_12000_Yes]	t(4)=0.50	p< 0.6433	t(4)=0.27	p< 0.8025	t(4)=0.86	p< 0.4384	t(4)=3.02	p< 0.0392
[HSM_12000_No]vs[HSM_12000_Yes]	t(4)=2.23	p< 0.0897	t(4)=2.17	p< 0.0955	t(4)=0.77	p< 0.4865	t(4)=0.57	p< 0.6004

PAIRWISE COMPARISONS [Q=TukeyHSD: *=p<0.05 **=p<0.01]
Sample description contains the following information: (mucus type)_(concentration)_(the presence of oseltamivir)
PBS 0 sample is denoted PSM_0 or HSM_0

Analysis summary

A/PR/8/34(H1N1)
ANOVA: Design 3 Between Subject Factors
mucus F(1,32) = 129 p<0.000001 SS=3.06 MSe=0.02
CONC F(3,32) = 17.9 p<0.000001 SS=1.28 MSe=0.02
INHIBITOR F(1,32) = 43.0 p<0.000001 SS=1.02 MSe=0.02
mucus*CONC F(3,32) = 15.0 p<0.000003 SS=1.07 MSe=0.02
mucus*INHIBITOR F(1,32) = 0.430 p<0.516487 SS=0.01 MSe=0.02
CONC*INHIBITOR F(3,32) = 0.542 p<0.657188 SS=0.04 MSe=0.02
mucus*CONC*INHIBITOR F(3,32) = 1.11 p<0.358579 SS=0.08 MSe=0.02

A/SD/1/2009(SOIV)
ANOVA: Design 3 Between Subject Factors
mucus F(1,32) = 40.9 p<0.000001 SS=0.87 MSe=0.02
concentration F(3,32) = 15.7 p<0.000002 SS=1.00 MSe=0.02
inhibitor F(1,32) = 189 p<0.000001 SS=4.02 MSe=0.02
mucus*concentration F(3,32) = 5.12 p<0.005272 SS=0.33 MSe=0.02
mucus*inhibitor F(1,32) = 4.69 p<0.037968 SS=0.10 MSe=0.02
concentration*inhibitor F(3,32) = 4.52 p<0.009392 SS=0.29 MSe=0.02
mucus*concentration*inhibitor F(3,32) = 1.40 p<0.259810 SS=0.09 MSe=0.02

A/SD/17/2008(H1N1)
ANOVA: Design 3 Between Subject Factors
mucus F(1,32) = 17.0 p<0.000251 SS=0.39 MSe=0.02
concentration F(3,32) = 11.6 p<0.000026 SS=0.79 MSe=0.02
inhibitor F(1,32) = 29.8 p<0.000005 SS=0.68 MSe=0.02
mucus*concentration F(3,32) = 3.09 p<0.040822 SS=0.21 MSe=0.02
mucus*inhibitor F(1,32) = 0.013 p<0.909212 SS=0.01 MSe=0.02
concentration*inhibitor F(3,32) = 0.551 p<0.651421 SS=0.04 MSe=0.02
mucus*concentration*inhibitor F(3,32) = 0.805 p<0.500577 SS=0.05 MSe=0.02

A/Aichi/2/68(H3N2)
ANOVA: Design 3 Between Subject Factors
Mucus F(1,32) = 80.0 p<0.000001 SS=3.59 MSe=0.04
Concentration F(3,32) = 7.39 p<0.000677 SS=1.00 MSe=0.04
Inhibitor F(1,32) = 3.98 p<0.054496 SS=0.18 MSe=0.04
Mucus*Concentration F(3,32) = 11.3 p<0.000032 SS=1.53 MSe=0.04
Mucus*Inhibitor F(1,32) = 0.008 p<0.929982 SS=0.01 MSe=0.04
Concentration*Inhibitor F(3,32) = 0.208 p<0.890387 SS=0.03 MSe=0.04
Mucus*Concentration*Inhibitor F(3,32) = 0.266 p<0.849203 SS=0.04 MSe=0.04
